# Supplementary material for: Intraductal Carcinoma of the Prostate: To Grade or Not to Grade
Source: Cancers (Basel). 2023 Nov 7;15(22):5319. doi: 10.3390/cancers15225319 (PMC10669759; doi:10.3390/cancers15225319)
Supplement: Supplementary file 1 [file cancers-15-05319-s001.zip › cancers-2666413-supplementary.pdf]

Supplemental Table S1: Grade Group discrepancy between GUPS and ISUP grading methods

**Authors, Year**	**Chen-Maxwell, 2020** [19]	**Rijstenberg, 2020** [18]		**Rizzo, 2021** [21]		**Tzelepi, 2021** [20]
**Type of specimen**	NBx	NBx	RP (entirely embedded)	NBx		RP (not entirely embedded)
**Definition of GG**	Global GG defined as the most common and the highest GG of all cores	Global GG (graded as if one long core)	-	Highest GG, global GG, and NCCN risk category		-
**GG included in the study**	GG1–4	GG1-5		GG1–4		GG1-5
**Usage of BCM IHC**	Yes, if basal cells are not clearly visible on H&E (IHC done in 83% of 123 cases)	Yes, if morphology cannot distinguish IDC-P and PCa		Yes, all foci suggestive of IDC-P lacking GG5 PCa		No, unless already available (already ordered at sign out)
**Total # of cases (cancer and benign)**	4630	N/A		N/A		N/A
**# of cancer cases**	2726 (59% of all NBx)	1031	835	48		129
**# of cases with IDC-P**	123 (IDC-P with GG1–4 PCa only)	139 (13.5%)	213 (25.5%)	48		81 /129 (63%)
**# of cases that GGs were discrepant**	28 (1% of positive NBx or 0.6% of all NBx)	17 (1.6% of positive NBx)	5 (0.6% of RP)	11 (22.9% of cases with IDC-P and invasive PCa) for highest GG	13 (27.1% of cases with IDC-P and invasive PCa) for global GG	2 (1.6% of all RP and 2.5% of RP with IDC-P)

**Difference in GG if IDC-P was graded**	Upgrading by 1–3 categories	Upgrading by 1 category	Upgrading by 1–2 categories	Upgrading by 1–4 categories	Upgrading by 1–4 categories; downgrading by 1 category in 1 case	Upgrading by 1 and 3 categories
**Comments**	- 4 NBx showed IDC-P with GG1 PCa<br>- 23% discordance is misleading without knowledge of the denominator	4 NBx showed IDC-P with GG1 PCa	2 RP showed IDC-P with GG1 PCa	- 3 NBx showed IDC-P with GG1 PCa<br>- Downgrading resulted from grading micropapillary IDC-P with pleomorphic nuclei as GP3<br>- Increased NCCN risk category by 1 or 3 steps in 4/11 (36.6%) cases due to increased GG when IDC-P was graded		No IDC-P with GG1 PCa

BCM, basal cell marker; GG, Grade Group; GP, Gleason pattern; GUPS, Genitourinary Pathology Society; IDC-P, intraductal carcinoma of the prostate; IHC, immunohistochemistry; ISUP, International Society of Urological Pathologists; N/A, not available; NBx, needle biopsy; NCCN, National Comprehensive Cancer Network; PCa, prostate cancer; RP, radical prostatectomy

Supplemental Table S2: Case reports of isolated IDC-P and IDC-P associated with GG1 PCa

**Authors, Year**	**Cohen, 2007** [22]	**Robinson, 2010** [23]	**Miyai, 2014** [24]	**Khani, 2015** [25]	**Khani, 2019** [26]	**Rijstenberg, 2020** [18]	**Grypari, 2020** [27]	**Ortiz-Rey, 2022** [28]
**Type of specimen**	RP	RP	RP	RP	RP	RP	RP	RP
**Prostate entirely submitted**	Not stated	Yes	No	No	In 53% of cases	Yes	1st case: 80%<br>2nd case: yes	Yes
**Concomitant PCa**	GS 3+2=5, not in close proximity to IDC-P	Isolated IDC-P	No PCa within 3 mm of IDC-P, or PCa present within 3 mm but very small (<0.05 mL) and showed transition from HGPIN to IDC-P	IDC-P with GG1 PCa	Isolated IDC-P and IDC-P with GG1 PCa	IDC-P with GG1 PCa	1st case: GG4 PCa away from IDC-P<br>2nd case: isolated IDC-P	IDC-P with GG1 PCa
**# of cases with possible precursor IDC-P**	1	2	14	3	15	2	2	2
**F/U time**	3 months	1 year	7 years	52 months	N/A	N/A	1st case: 40 months<br>2nd case: no F/U	34 and 2 months
**Outcome**	No BCR	Disease free	No BCR in 93%	1 BCR	N/A	N/A	No BCR	No BCR

**Remarks**	NBx showed IDC-P in 16/17 cores	NBx showed pure IDC-P	-	NBx showed IDC-P with GG1 PCa	A subset showed discordant PTEN/ERG expression and *MAPK/PI3K* mutation	-	-	-
BCR, biochemical recurrence; ERG, ETS-related gene; F/U, follow-up; GG, Grade Group; GS, Gleason score; HGPIN, high-grade prostatic intraepithelial neoplasia; IDC-P, intraductal carcinoma of the prostate; *MAPK*, mitogen-activated protein kinase; N/A, not available; NBx, needle biopsy; PCa, prostate cancer; *PI3K*, phosphoinositide 3-kinase; PTEN, phosphatase and tensin homolog; RP, radical prostatectomy								
